# Genotype-phenotype matching analysis of 38 *Lactococcus lactis* strains using random forest methods

**DOI:** 10.1186/1471-2180-13-68

**Published:** 2013-03-26

**Authors:** Jumamurat R Bayjanov, Marjo JC Starrenburg, Marijke R van der Sijde, Roland J Siezen, Sacha AFT van Hijum

**Affiliations:** 1 Centre for Molecular and Biomolecular Informatics, Radboud University Medical Centre, PO Box 9101, Nijmegen, The Netherlands; 2 Netherlands Bioinformatics Centre, 260 NBIC, P.O. Box 9101, Nijmegen 6500 HB, The Netherlands; 3 NIZO Food Research, P.O. Box 20, BA Ede 6710, The Netherlands; 4 TI Food and Nutrition, P.O. Box 557, Wageningen 6700 AN, The Netherlands; 5 Kluyver Centre for Genomics of Industrial Fermentation, P.O. Box 5057, Delft, GA 2600, The Netherlands; 6 Present address: Department of Genetics, University Medical Center Groningen, University of Groningen, Hanzeplein 1, Groningen 9713 GZ, The Netherlands

## Abstract

**Background:**

*Lactococcus lactis* is used in dairy food fermentation and for the efficient production of industrially relevant enzymes. The genome content and different phenotypes have been determined for multiple *L. lactis* strains in order to understand intra-species genotype and phenotype diversity and annotate gene functions. In this study, we identified relations between gene presence and a collection of 207 phenotypes across 38 *L. lactis* strains of dairy and plant origin. Gene occurrence and phenotype data were used in an iterative gene selection procedure, based on the Random Forest algorithm, to identify genotype-phenotype relations.

**Results:**

A total of 1388 gene-phenotype relations were found, of which some confirmed known gene-phenotype relations, such as the importance of arabinose utilization genes only for strains of plant origin. We also identified a gene cluster related to growth on melibiose, a plant disaccharide; this cluster is present only in melibiose-positive strains and can be used as a genetic marker in trait improvement. Additionally, several novel gene-phenotype relations were uncovered, for instance, genes related to arsenite resistance or arginine metabolism.

**Conclusions:**

Our results indicate that genotype-phenotype matching by integrating large data sets provides the possibility to identify gene-phenotype relations, possibly improve gene function annotation and identified relations can be used for screening bacterial culture collections for desired phenotypes. In addition to all gene-phenotype relations, we also provide coherent phenotype data for 38 *Lactococcus* strains assessed in 207 different phenotyping experiments, which to our knowledge is the largest to date for the *Lactococcus lactis* species.

## Background

*Lactococcus lactis* – a low-GC Gram-positive model organism, found frequently in both dairy and non-dairy [1] environments, has been extensively studied due to its industrial importance. Major focus of these studies has been on dairy isolates, of which the genomes of three isolates have been sequenced [2-4]. Plant isolates compared to dairy isolates show higher stress-tolerance and have more extensive fermentative abilities [5]. Due to their larger genetic and metabolic repertoire non-dairy isolates of *L. lactis* are therefore of interest in dairy food fermentation [6]. Strains used in dairy starter cultures have presumably evolved from plant strains, where some metabolic capabilities were lost in order to adapt to dairy environments [7]. Recently, the genome of ssp. *lactis* strain KF147 was fully sequenced [8] and that of strain KF282 was partially sequenced [9]. These two plant *L. lactis* isolates were reported to possess many genes related to uptake of plant cell-wall degradation products such as arabinose and xylose [9]. Many genes present in these two isolates are new and do not have homologs in the three *L. lactis* strains IL1403, MG1363 and SK11 of dairy origin [9]. Recently, the genomes of several other *L. lactis* strains have also been fully sequenced [10-13]. Furthermore, many *L. lactis* strains were reported to have plasmids, enriching the genotypic and phenotypic repertoire of this species [3,14]. *L. lactis* strains isolated from different niches have been reported to have high genomic sequence divergence [15-17], also at the subspecies level [18]. Their gene content partly reflects their phenotypic properties such as niche adaptation [9,16,18].

In general, genomic and phenotypic properties of strains have been studied separately [19,20], and less frequently possible relations between genes and phenotypes have been studied [21]. Integrative genotype-phenotype matching would facilitate identifying genetic markers relevant for the manifestation of a phenotype. We therefore used an iterative gene selection procedure coined PhenoLink [22] to more accurately determine gene to phenotype relations of 38 *L. lactis* strains from 3 different subspecies: ssp. *lactis*, ssp. *cremoris* and ssp. *hordniae* (see Table 1). This allowed identifying novel gene-phenotype relations as well as confirming previously reported relations. In addition to identified gene-phenotype relations, we also present a coherent dataset of genotype and phenotype data based on 207 experiments, which could prove to be valuable in comparative analysis of these strains.

**Table 1:** **Genotype and phenotype information for 38 *L.lactis *strains that was used in genotype-phenotype matching**

**Strain name**	**Subspecies**	**Isolation origin**	**# present genes (out of 4026)**	**# phenotyping experiments (out of 130**^**a**^**)**
AM2	*cremoris*	dairy	2563	119
ATCC19435T	*lactis*	dairy	2047	121
DRA4	*lactis*	dairy	2182	123
E34	*lactis*	plant	2022	123
FG2	*cremoris*	dairy	2301	117
HP	*cremoris*	dairy	2307	122
IL1403	*lactis*	dairy	2289	127
K231	*lactis*	plant	2067	124
K337	*lactis*	plant	2002	126
KF134	*lactis*	plant	2039	128
KF146	*lactis*	plant	2087	130
KF147	*lactis*	plant	2472	126
KF196	*lactis*	plant	1978	126
KF201	*lactis*	plant	2020	125
KF24	*lactis*	plant	2119	128
KF282	*lactis*	plant	1937	127
KF67	*lactis*	plant	2096	128
KF7	*lactis*	plant	2109	125
KW10	*cremoris*	plant	2039	126
LMG14418	*lactis*	dairy	2259	113
LMG6897T	*cremoris*	dairy	2308	113
LMG8520	*hordniae*	insect	1903	113
LMG8526	*lactis*	plant	1985	123
LMG9446	*lactis*	plant	1983	125
LMG9449	*lactis*	plant	2221	125
Li-1	*lactis*	plant	2198	126
M20	*lactis*	plant	2090	121
MG1363	*cremoris*	dairy	2397	125
ML8	*lactis*	dairy	2339	123
N41	*cremoris*	plant	2405	121
N42	*lactis*	plant	2361	125
NCDO763	*cremoris*	dairy	2414	126
NCDO895	*lactis*	dairy	2285	124
P7266	*lactis*	plant	1917	126
P7304	*lactis*	plant	2223	127
SK11	*cremoris*	dairy	2551	119
UC317	*lactis*	dairy	2280	125
V4	*cremoris*	dairy	2313	113

**a**: In total there are 207 phenotyping experiments (see Additional file 1), but only 130 were usable in our analysis (see Results).

## Results

### Strain similarity based on phenotypes

A recent extensive genotyping study of *L. lactis* strains revealed that clustering based on chromosomal genes of these strains shows a high correspondence with the sub-speciation, whereas clustering using plasmid genes reflects niche-adaptation properties [16]. In this study, we also analyzed these strains using only their phenotypic measurements in 207 experiments (Additional file 1). The used phenotypic metrics differ depending on the type of experiment performed. Using all phenotypic measurements in clustering could result in clusters that consist of phenotypic measurements that are in fact incomparable, for example, phenotypic readout of 2 in an API test indicates no growth, whereas the same value obtained in the GM17 medium shows growth (see Additional file 1). From the phenotype clustering, where pre-processed phenotype data was used, we conclude that only some phenotype types partly co-cluster (for instance metal resistance; bottom part of phenotype-based clustering dendrogram as shown in Additional file 2). However the phenotype grouping is not very apparent from clustering phenotypic measurements only. To this end, five categories of experiments were defined based on experiment type: (i) growth on sugars, (ii) antibiotic resistance, (iii) metal resistance, (iv) growth on milk or polysaccharides and (v) remaining experiments (see also Table 2 and Additional file 1). Visualization of links to all phenotypes creates a very large figure that is difficult to present and interpret (results not shown). Since each experiment category represents a related set of experiments, each experiment category was analyzed separately. Therefore for four of the experiment categories (Table 2), strains were hierarchically clustered based on their phenotypes (see phenotype clustering section of the Additional file 2). Based on the hierarchical clustering results, strains isolated from the same source showed different levels of phenotype similarity: growth on sugar (high similarity), antibiotic resistance experiments (medium similarity), growth on milk and polysaccharides (low similarity) and metal resistance (no similarity). Phenotype-based hierarchical clustering of these strains showed that niche properties better correspond to phenotype differences of strains rather than their subspecies-level differences. Clustering provided only limited information and, thus, it can only be used as an initial screening of phenotype data. As the focus of this study is to find relations between genes and phenotypes we applied integrative analysis of phenotype and genotype data to reveal these associations.

**Table 2:** Experiments grouped based on experimental conditions

**Group name**	**Number of experiments**	**Description**
Growth on sugar	16	Contains phenotypes based on 50CH API experiments
Antibiotic resistance	18	Contains phenotypes based on antibiotic resistance experiments
Metal resistance	17	Contains phenotypes based on metal resistance experiments
Growth on milk or polysaccharides	11	Contains phenotypes based on growth on milk or polysaccharides
Other experiments	10	Contains phenotypes based on all remaining experiments, which include growth test on medium with nisin, arginine hydrolase, salt or different enzymes.

These are experiments of which at least a single phenotype was accurately classified; for full list of experiments and their descriptions see Additional file 1.

### Genotype-phenotype matching

Integrated analysis using an iterative gene selection allowed identification of gene-phenotype relations that could not be found by studying genotype and phenotype data separately. In genotype-phenotype matching, we used the presence/absence of 4026 ortholog groups (OGs; see Methods) in 38 *L. lactis* strains (Table 1) determined by comparative genome hybridization (CGH) as genotype data. These 38 strains are a subset of a large representative collection of *L. lactis* trains that covers genotype, niche and phenotype diversity of *L. lactis* species [15]. For phenotype data, we used phenotypic measurements of these strains in 207 experiments that were previously assessed in separate studies (see Methods and Additional file 1). After pre-processing, phenotype data from 130 experiments was usable for genotype-phenotype matching (see Methods). Only associations of genes to accurately classified phenotypes (see Methods) were considered in further analysis, which resulted in 140 phenotypes, assessed in 74 different experiments.

Many gene-phenotype relations were identified: a total of 1388 OGs or on average 565 genes per reference strain were identified to be related to at least one of these 140 phenotypes. In the present study, we focussed on gene clusters consisting of at least two phenotype-related genes that are in close genomic proximity (e.g., in operons; see Methods). Transposases, integrases and phage proteins were also removed, because relations between these proteins and phenotypes are likely to be spurious. Discarding above-mentioned genes decreased the percentage of phenotype-related genes by about 50% on average. In analyzing gene clusters, we first considered gene clusters of which their presence relates to a positive trait (e.g., growth) and absence relates to a negative trait (e.g., no growth). There were also many gene clusters with inverse patterns, where an absence of a gene cluster leads to a positive trait. An inverse relationship between genes and phenotypes might indicate that in the absence of a regulator, genes previously inhibited by this particular regulator can become active, which in turn might lead to a positive trait (e.g., survival of a strain). In the supplementary data we provide all identified relations including inverse relations (see genotype-phenotype relations in an Additional file 2 that contains a mini-website).

### Genes related to carbohydrate utilization

**Figure 1:**
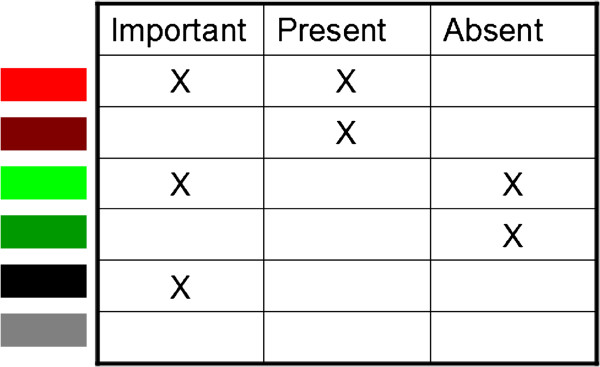
**Integration of gene significance with its presence/absence.** A gene that is present in at least 75% of strains of a phenotype is assumed to be predominantly present and a gene that is absent in at least 75% of strains of a phenotype is assumed to be predominantly absent; otherwise a gene is assumed to be present in a subset of strains. Gene-phenotype relations were visualized by integrating each gene’s phenotype importance with its predominant presence/absence in strains of this particular phenotype, whereas in visualizing gene-strain relations gene’s contribution score and presence/absence in a corresponding strain were used.

**Figure 2:**
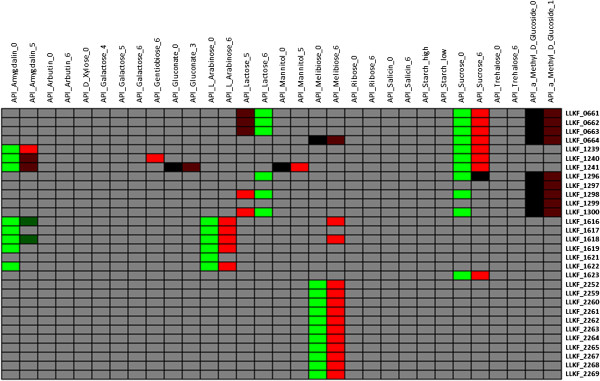
***L. lactis *KF147 gene clusters correlated to growth on the sugars arabinose, melibiose and sucrose.** Colours represent strength of relationship between a gene and a phenotype (Figure 1). Phenotypes are either shown as last digits in column names or with suffixes “high” or “low”, where 0 indicates there is no growth and other numbers indicate different growth levels in different experiments as described in the Additional file 1. Here “high” and “low” phenotypes indicate high and low growth levels, respectively. For gene annotations see Additional file 3.

Several gene clusters related to fermentation of different sugars were identified by genotype-phenotype matching. Among them were gene clusters that were previously described to be involved in carbohydrate utilization [16]. For instance, the presence of a gene cluster required for arabinose utilization [9] was confirmed in this study to correlate strongly with the ability to grow on arabinose (see Figure 1 for colour-coded representation of gene-phenotype relations and Figure 2 for gene-phenotype relations of KF147 genes LLKF_1616-1622, and their orthologs in query strains). Several gene clusters were found to be related to sucrose utilization; for instance a cluster of 4 genes (LLKF_0661-LLKF_0664 in strain KF147, and their orthologs in query strains) that already was annotated as being involved in sucrose utilization (Figure 2) [8]. The other three reference strains do not grow on sucrose, and this gene cluster was absent in these strains. These genes were also found to be inversely related to growth on lactose, where they were present in most of the strains that grew slowly on lactose and absent in most of the strains that can grow on lactose (Figure 2). Such a relationship suggests that most of the strains that grow well on sucrose (22 strains) cannot grow or grow slowly on lactose (17 out of 22 strains) or vice-versa (10 out of 15 lactose-degrading strains cannot grow on sucrose). This also partly reflects niche adaptation of these strains, because most of the lactose-degrading strains were dairy isolates (10 out of 15) and most of the sucrose-utilizing strains were of plant origin (17 out of 22). Additionally, we also identified an association between sucrose fermentation and nisin production in *L. lactis*. Both sucrose utilization and nisin biosynthesis genes were earlier reported to be encoded on a transposon in strain NIZO R5 [23]. Additionally, linkage between these phenotypes has been observed in 13 *L. lactis* strains [24]. Visualization of identified gene-phenotype relations revealed that sucrose-negative strains lack part or all of the genes related to nisin production. For example, KF147 - a nisin non-producer strain - contains only part of the nisin gene cluster, conferring immunity but not production (see LLKF_1296, LLKF_1298 and LLKF_1300 in Figure 2) [9]. However, we found no strong relation between growth on sucrose and presence of nisin biosynthesis genes, confirming a previous observation that the presence of nisin biosynthesis genes in a strain does not always confer its growth on sucrose [25].

A large cluster of 11 genes (Figure 2) was found to be related to growth on melibiose, a plant disaccharide, but not to any of the other carbohydrates tested. This confirms an earlier observation that strain KF147 can utilize this disaccharide while 3 other strains IL1403 (dairy), SK11 (dairy) and KF282 (plant) strains cannot grow on melibiose [9,26]. We also investigated whether a genomic region that encompasses these genes was deleted in melibiose-negative strains, because chromosomal deletion of a 12 kb region in *Streptococcus mutans* strains leads to melibiose-negative phenotype [27,28]; this 12 kb region contains orthologs of LLKF_2260-2262 of strain KF147. Because tiling pan-genome CGH arrays were used to identify gene occurrence in these strains, deletion of a genomic region in query strains can be determined (see Methods). Therefore, we visualized a small genomic region of approximately 20 Kb (see Additional file 2) that covers the starting position of LLKF_2250 and the end position of LLKF_2270 on the KF147 genome. This region encompasses all these 11 genes and several more genes. Indeed, we also observed that this large 20 Kb region was deleted or absent in all melibiose-negative strains from both plant and dairy origin (see Additional file 2). Probably, only 10 genes consecutively located in a 15 Kb region (corresponding to genes LLKF_2259-LLKF_2269 in strain KF147) are necessary for growth on melibiose.

### Genes related to metal resistance

**Figure 3:**
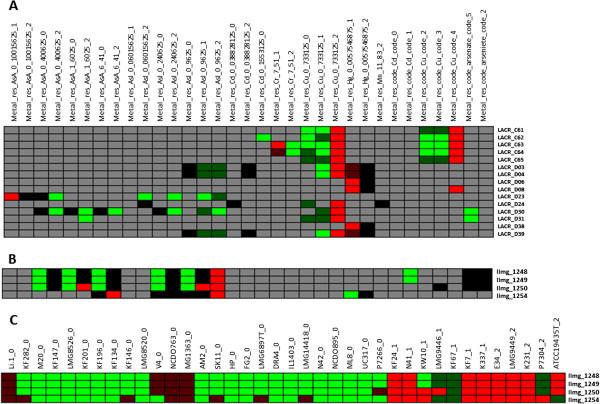
**Genes related to metal resistance. A**) Genes correlated to copper resistance were found on plasmids C and D of *L. lactis* SK11. **B**) *L. lactis* MG1363 genes that were found to be correlated to arsenite resistance. **C**) Gene-to-strain relations for *L. lactis* MG1363 genes shown in B. Colours represent strength of relationship (Figure 1) between a gene and a phenotype for A and B, but between a gene and a strain for C. Phenotypes are shown as the final digits in column names, where 0 indicates there is no resistance and other numbers indicate different resistance levels in different experiments as described in the Additional file 1. For gene annotations see Additional file 3.

Using genotype-phenotype matching several gene clusters were found relating to heavy metal resistance, and some of these genes are located on plasmids. For instance, we found clusters of genes related to copper resistance; these are located on plasmids C and D in strain SK11 (Figure 3A), which confirms a previous finding [29]. One of these gene clusters (LACR C61-C65 in strain SK11, and their orthologs in query strains) was previously identified to be involved in copper resistance [14]. Additionally, a cluster of four genes (llmg1248-1250, llmg_1254 in strain MG1363, and their orthologs in query strains) was identified by gene-trait matching to be related to arsenite resistance (Figure 3B and 3C), which is usually known as a plasmid-borne trait [29], and two of these genes are annotated as arsenical-resistance proteins (Additional file 3). However, these could be plasmid genes that were transferred to the chromosome in the plasmid curing process of MG1363.

### Genes related to arginine metabolism

**Figure 4:**
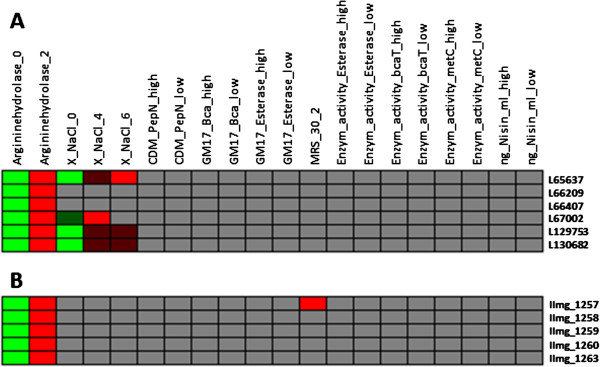
**Genes related to arginine metabolism. A**) Two clusters of *L. lactis* IL1403 genes related to arginine metabolism. **B**) A *L. lactis* MG1363 gene cluster correlated to arginine metabolism. Colours represent strength of relationship between a gene and a phenotype (Figure 1). Phenotypes are either shown as last digits in column names or with suffixes “high” or “low”, where 0 indicates no growth and other numbers indicate different growth levels as described in the Additional file 1. Here “high” and “low” phenotypes indicate high and low enzyme activity levels, respectively. For gene annotations see Additional file 3.

Several gene clusters were found to be relevant to arginine hydrolase activity, and therefore the ability to metabolize arginine. A cluster of 4 genes (L65637, L66209, L66407 and L67002 in strain IL1403, and their orthologs) was identified to be relevant to arginine metabolism (Figure 4A). All 4 proteins are annotated as hypothetical proteins in strain IL1403 and two of them, L66209 and L67002, are probably membrane proteins as they belong to a cluster of orthologous groups of proteins (COGs) [30], which contains membrane proteins. A gene cluster of 5 MG1363 genes was also identified to be related to arginine metabolism (Figure 4B), and two encoded proteins, llmg_1257 and llmg_1259, are in the same COGs with proteins L66209 and L67002 of strain IL1403. The protein L67002 belongs to a family of membrane proteins of which some are glycosyltransferase-associated proteins. Probably, at least two of these proteins, L66209 and L67002, and their MG1363 orthologs, llmg_1257 and llmg_1259, should be re-annotated as transport proteins or maybe more specifically arginine transport proteins. However, experimental validation is necessary.

### Plasmid genes related to phenotypes

**Figure 5:**
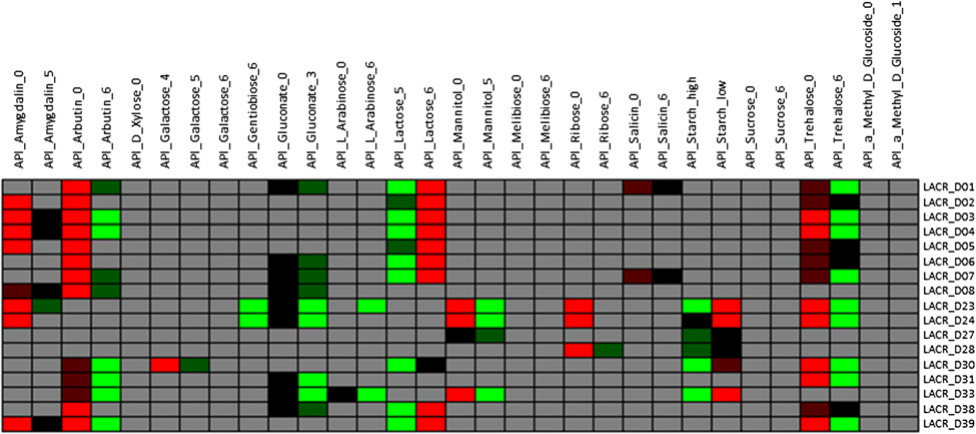
**Genes correlated to growth on lactose were found on plasmid D of *L. lactis *SK11.** Colours represent strength of relationship between a gene and a phenotype (Figure 1). Phenotypes are either shown as last digits in column names or with suffixes “high” or “low”, where 0 indicates there is no growth and other numbers indicate different growth levels in different experiments as described in the Additional file 1. Here “high” and “low” phenotypes indicate high and low growth levels, respectively. For gene annotations see Additional file 3.

Plasmid genes are necessary for manifestation of some phenotypes. For instance, it is already well-known that the lactose metabolism genes are localized on plasmid D of SK11 [14]. Indeed, we found that the presence/absence of these lactose metabolism genes (LACR_D01-07 and LACR_D38-39 in SK11, and their orthologs in query strains) in the 38 strains to be highly correlated to growth on lactose (Figure 5). Again, there appears to be an inverse relationship with the presence of these same lactose utilization genes for no-growth on some other sugars (trehalose, arbutin, amygdalin). Thus, using plasmid genes in addition to chromosomal genes in genotype-phenotype matching allowed confirming previously known functions of some plasmid genes and identifying novel relationships between plasmid genes and some phenotypes.

### Partial gene-phenotype relations

For each experiment category several (on average 9) partial relations between gene clusters and phenotypes, where a gene is present in only a subset of strains with a particular phenotype (Figure 1), were identified. Most of these gene clusters contain only two genes and were often found to be relevant to a negative trait (e.g.: no-growth). As example we present partial relations between a cluster of four genes of strain MG1363 (and their orthologs in query strains) and arsenite resistance (Figure 3B). These genes were found to be relevant for strains growing at 0.9625 mM of arsenite and are present in most of the highly resistant strains. However, some of these genes are only present in a subset of strains with no or mild resistance (Figure 3B). Visualizing occurrence of these genes in strains revealed that they are mostly absent in strains with no arsenite resistance phenotype and mostly present in strains with mild or high arsenite resistance phenotypes (Figure 3C).

## Discussion

Genotype-phenotype association analysis of 38 *L. lactis* strains by integrating large genotype and phenotype data sets allowed screening of gene to phenotype relations. Only the top 50 genes per phenotype were selected as important (see Methods), because probably most relevant genes related to a phenotype should be among these 50 genes and their correlated genes. Indeed, only less than 1% of phenotypes had 50 or more related genes in the top list. Furthermore, identified relations were visualized by integrating each gene’s occurrence with its phenotype importance, which allows a quick screening of many relations. However, some relations could be due to an indirect effect of other factors that were not taken into account. For example, the anti-correlation between sucrose and lactose metabolism could be a bias resulting from starter-culture selection programmes, where often bacteriocin-negative strains were selected that could have led to selection of strains that can use lactose instead of sucrose. Additionally, for some phenotypes we could not find many related genes, for example, well-known arginine-metabolism related genes were not found as relevant to metabolism of arginine. Therefore, we analyzed all OGs with gene members containing a word ‘arginine’ in their annotation and genes of the arginine deiminase pathway (*arcABCD*). However, all these genes were either present in all or in at least 36 out of 38 strains, and such genes are removed in the pre-processing step of PhenoLink, because they are not capable to separate strains with different phenotypes (see Methods). We described a few examples where the annotation of genes could be refined and a few cases where new functions are suggested for genes with unknown functions. We were able to pinpoint only a few novel relations, but analyzing all identified gene-phenotype relations in detail should allow finding even more novel relations and refining annotations of more genes.

Genotype-phenotype matching allows comprehensive screening for possible relations between genes and phenotypes. We had data for 38 strains and, thus, there were relatively few strains with a given phenotype and in some experiments many strains manifested the same phenotype. Therefore, few partial gene-phenotype relations were identified in this study. More partial relations could be identified using data sets based on more *L. lactis* strains, which would allow finding analogous genes that have similar function but different sequences.

Even with DNA sequencing prices dropping, determining the gene content of dozens of strains by genome sequencing could still be costly. Pan-genome arrays allow querying occurrence of genes in multiple strains more cost-effectively, but genes absent in reference sequences and strongly divergent genes would be missed. Though the presence/absence data can be linked to phenotypes, it cannot account for effects of regulatory control or post-translational modifications. Thus putative gene-phenotype relations should be experimentally tested by high-throughput techniques such as gene expression analysis.

Annotating genes of a genome is essential in understanding the genomic properties of any strain. Gene annotation is often based on sequence similarity, so mistakes in annotating a single gene could propagate to genes of different organisms through annotation by sequence similarity. Therefore identified gene-phenotype relations should be experimentally validated and linked to other information sources such as pathway information. This would allow decreasing error propagation introduced by sequence similarity based gene function prediction approaches. Genotype-phenotype matching results show that the largest group of proteins related to phenotypes was hypothetical proteins indicating that gene annotations could still be improved for all 4 reference strains. Genomes of more bacterial strains are sequenced on a daily basis, which shows the critical importance of accurate gene function prediction. Identified gene-phenotype relations would allow more accurately determining functions of many genes, and hence better understanding of genotype- and phenotype-level differences among 38 *L. lactis* strains. We provide all identified relations as well as complete genotype and phenotype data set (see Additional files). This data set not only serves as a collection of leads to phenotypes, but due to large data size could also be used to test different association methods.

## Conclusions

*Lactococcus lactis* has been extensively studied due to its industrial importance. Here we provide a coherent genotype and phenotype dataset and its interpretation for the *Lactococcus* species. We integrated for 38 *L. lactis* strains their genotypic measurements as well as phenotypes derived from 207 different experiments (see Methods) to identify gene-phenotype relations. Our results are publicly available (see also Additional files) and contains many leads into *Lactococcus* species-wide genotype-phenotype relations that can further be analysed and experimentally validated. These relations could be used to refine functions of genes. As new genome sequences emerge frequently, this would allow annotating gene functions for these new genomes more accurately and predicting phenotypes of new strains based on their DNA sequence.

## Methods

### Strains

For genotyping, a total of 39 *L. lactis* strains were selected from 91 *L. lactis* strains of which several phenotype and genotype properties were previously assessed [15]. These strains were isolated from plant and dairy niches and belong to 3 different subspecies: *lactis* (28 strains), *cremoris* (10 strains) and *hordniae* (one strain). These strains represent the genotype, niche and phenotype diversity of the *L. lactis* species [15]. Phenotypic properties of the strain NIZOB2244B were not assessed; therefore, 38 strains were used in genotype-phenotype matching (see Table 1).

### Phenotypic diversity tests

Strains were incubated in 96-well micro-plates in quadruplicate in 250 μl M17 broth (Oxoid Ltd., Basingstoke, Hampshire, England) supplemented with 1% glucose (wt/vol) (GM17). Medium was supplemented either with different concentrations of NaCl; nisin (Sigma Chemical, St Louis, USA); metals; antibiotics; or polysaccharides (see Additional file 1). The plates were incubated overnight at 30°C [31].

For incubation of strains in GM17 medium different temperatures (4, 17, 30, 37 or 45°C) were used. Strains were also incubated in several other media: skimmed milk, skimmed milk supplemented with 0.5% yeast extract (Difco, Becton, Dickinson and company, Sparks, USA) and MRS-broth (Merck KGaA, Germany). Fermentation tests of arginine hydrolase activity, 50 different sugars and citrate were performed as reported previously [15]. Activity of several enzymes, i.e. branched chain aminotransferase, alpha-hydroxyisocaproic acid dehydrogenase, aminopeptidase N, cystathionine β lyase, X-prolyl dipeptidyl aminopeptidase and esterase in strains growing on GM17-broth or CDM-media, were previously assessed [32,33]. More information about phenotyping experiments and results of these experiments are available in an Additional file 1.

### Genotype data

The gene content of *L. lactis* strains was previously determined by pan-genome CGH arrays, where tiling array probes were based on chromosomal, plasmid and single gene or operon DNA sequences of this species as described in [34]. Next to probes targeting all known genes within *Lactococcus* sp. [35] we additionally targeted intergenic regions. However, in this study, we did not use the probes targeting intergenic regions. We grouped orthologous genes into ortholog groups (OGs); bidirectional orthologous relations among genes of four fully sequenced strains were identified by pair-wise comparisons using InParanoid [36] with default parameters [34]. The genomes used were from *L. lactis* strains *ssp. lactis* IL1403, *ssp. lactis* KF147, *ssp. cremoris* SK11 and *ssp. cremoris* MG1363. MG1363 replaces the incomplete chromosomal sequence of KF282 strain that was used in the array design [34]. Genes with inconsistent bidirectional orthologous relations and plasmid genes of plasmid-containing strains (SK11 and KF147) were each treated as a separate OG containing a single gene. In total, 4026 OGs were created of which 149 specified single plasmid genes. For a gene member of an OG scoring the signal intensities of aligned probes determines its presence/absence in a query strain [34]. We used the PanCGHweb web-tool to find presence/absence of OGs in these strains [37].

### Visualizing and identifying presence or absence of a genomic segment

Presence or absence of contiguously located genes (i.e. a gene cluster) in a query strain indicates that the whole genomic region encompassing these genes is present or absent in this particular strain. Therefore presence or absence of a genomic segment in a query strain compared to a reference strain was identified. To this end, probes aligning to a genomic region of interest in a reference strain were identified. The log ratio of probe signals in a query strain to the reference strain was visualized to identify presence or absence of a genomic region in a query strain.

### Data pre-processing

In PhenoLink, genotype and phenotype data are pre-processed before using them in genotype-phenotype matching analysis. PhenoLink is based on the Random Forest algorithm [38]. In random forest classification, trees are trained based on random selections of genes and strains, genes with the same occurrence pattern could get different contribution scores [39]. This score is an estimate of how important a gene is to correctly classify a certain strain. Additionally, genes that are either present or absent in (almost) all queried strains have negligible impacts to separate strains of differing phenotypes [40]. Thus we did not use genes with homogeneous occurrence patterns and used only one of the highly correlated genes in further analysis. Prior to classification, phenotypes with continuous measurements were grouped into 3 bins, where each bin represents a different category. Strains that belong to the middle category were not used in genotype-phenotype matching to improve the classification accuracy. Additionally, in some experiments most of the strains exhibited a single phenotype such as the capability to grow on a certain sugar. Such an imbalance often leads to biased classification. Therefore imbalance in the number of strains per phenotype was decreased by creating 100 bags [22].

### Genotype-phenotype matching

Genes related to phenotypes were identified using PhenoLink mostly with default parameter settings. To decrease effects of random selection, the same genotype and phenotype data were classified 3 times and only genes consistently relating to phenotypes were selected. Additionally, only genes with a positive contribution score for at least a few (in this study 3) strains of a phenotype were used for further classification, which decreases spurious relations between genes and phenotypes. This iterative removal of genes continued until no more than a few (in this study 5) genes were removed [22]. Only relations to phenotypes that were classified with at least 60% accuracy were used in further visualization and analysis, which was empirically defined to allow visualizing even weaker relations. The accuracy was estimated by the Random Forest algorithm and is the percentage of strains that were correctly classified. For each phenotype, genes were sorted based on their phenotype importance, which is the sum of gene’s contribution score for each strain of this particular phenotype, and genes with the highest phenotype importance (in this study the top 50 genes) were selected. Genes that had homogenous occurrence patterns (variance < 0.05) were not used in genotype-phenotype matching. Highly correlated genes (e.g. members of the same operon) were added to the selected top genes provided that they were correlated to any gene in the top genes. The added gene was assigned the same phenotype importance as the gene to which it is correlated.

### Visualization of gene-phenotype relations

Visualization of the identified gene-phenotype relations facilitates quick screening and simplifies the analysis of these relations. Visualizing relations between accurately classified phenotypes (in this study a total of 140) and genes (here a total of 1388 OGs or on average 565 genes for each of the 4 reference strains) creates a large figure, which is difficult to analyze. To simplify visualization and analysis of gene-phenotype relations, phenotyping experiments were categorized into 5 groups based on experiment type: (i) growth on sugar, (ii) antibiotic resistance, (iii) metal resistance, (iv) growth on milk or polysaccharides and (v) remaining experiments (see also Table 2 and Additional file 1). Genes related to these phenotypes were visualized by merging the presence/absence of a gene with its phenotype importance. Since a gene’s presence/absence is strain-specific, its occurrence in strains of a phenotype was quantified to determine if a gene is predominantly present or absent. Merging predominant presence/absence of a gene with its phenotype importance creates 6 possible combinations each represented with a different colour as shown in Figure 1. A gene that is present in at least 75% of strains of a phenotype is assumed to be predominantly present and a gene that is absent in at least 75% of strains of a phenotype is assumed to be predominantly absent; otherwise a gene is assumed to be present in a subset of strains.

Visualization of gene-phenotype relations in reference strains allows identification of genes that are localized in close genomic proximity (e.g., members of the same operon). Therefore, gene-phenotype relations for corresponding genes of the reference strains were included in the visualization (see also Additional file 2). Two reference strains (SK11 and KF147) have plasmids; therefore, in the visualization a total of 149 plasmid genes were also used. In visualizing gene-phenotype relations, the phenotype importance of an OG was used for all its members. For each reference strain on average 565 gene-phenotype relations were found, but we focussed our analysis on phenotype-related gene clusters, which are genes in close genomic proximity. Two genes were considered in close proximity if a distance between their genomic starting positions did not exceed 2500 nucleotides, which was empirically determined. Using distances larger than 2500 nucleotides results in visualizing more non-neighbouring genes (false-positives), but using smaller distance would discard some neighbouring genes (false-negatives). Discarding true neighbours from visualization has more impact than including non-neighbours, because non-neighbouring genes can be easily recognized in visualization. Remaining gene-phenotype relations were visualized based on genomic order of genes.

Partial relations between genes and phenotypes, where a gene is present in only a subset of strains with a particular phenotype, were visualized with black colour (Figure 1). Gene’s occurrence in a strain was merged with its contribution score as shown in Figure 1. Gene-strain relations were visualized to show in which strains a gene is present and to which strains of a phenotype a gene was found to be relevant.

### Clustering of strains based on phenotypes

Hierarchical clustering of strains based on their phenotypes could reveal the phenotypic similarity of strains, which might be linked to their genotype. Thus, strains were hierarchically clustered based on the phenotypes using the *euclidean* distance metric and the *average* linkage agglomerative clustering method [39]. Experiments that only contained phenotype information for all 38 strains were used in clustering and strains were clustered for each of the 5 experiment categories separately (see Table 2 and Additional file 1). Clustering was not performed for fifth experiment category, because there were only 5 experiments where all 38 strains had phenotype information.

## Availability of supporting data

The data sets supporting the results of this article are included within the article and its additional files.

## Competing interests

The author declared that they have no competing interest.

## Authors’ contributions

JRB carried out genotype-phenotype association analysis and drafted the manuscript. MJCS carried out phenotypic tests. MRS is involved in genotype-phenotype analysis. RJS and SAFTH conceived of the study and drafted the manuscript. All authors read and approved the final manuscript.

## Supplementary Material

Additional file 1 **Phenotype data.** This file contains all phenotype used in this study and the file can be viewed with Microsoft Excel. Click here for file

Additional file 2 **Mini web-site that contains all figures generated in this study.** This mini web-site contains all figures of genotype-phenotype, projection and phenotype clustering results. Click here for file

Additional file 3 **Annotations for genes presented in gene-phenotype relations as shown in Figures** 2**–**5**.** This file contains gene annotations for genes that were shown in Figures 2–5 and the file can be viewed with Microsoft Excel. Click here for file
