# Supplementary material for: Genotype-phenotype matching analysis of 38 Lactococcus lactis strains using random forest methods
Source: BMC Microbiol. 2013 Mar 26;13:68. doi: 10.1186/1471-2180-13-68 (PMC3637802; doi:10.1186/1471-2180-13-68)
Supplement: Additional file 2 — Mini web-site that contains all figures generated in this study. This mini web-site contains all figures of genotype-phenotype, projection and phenotype clustering results. [file 1471-2180-13-68-S2.zip › Bayjanovetal_2012_Lactis/index.html]

|  |  |
| --- | --- |
| Description | Additional information |
| Gene-phenotype relations | Visualization of gene-phenotype relations for each of the 4 reference strains and for each of the 5 experiment groups in an HTML format. Because it is more easily browsable than separately analyzing each of the 20 figures. |
| Projection plots | Projection plots in an HTML page for easier browsing. |
| Phenotype clustering | Phenotype-based clustering of strains in an HTML page for easier browsing. |
| Phenotype and strain clustering | Clustering of phenotypes and phenotype-based clustering of strains in an HTML page for easier browsing. |
