# Supplementary material for: Genotype-phenotype matching analysis of 38 Lactococcus lactis strains using random forest methods
Source: BMC Microbiol. 2013 Mar 26;13:68. doi: 10.1186/1471-2180-13-68 (PMC3637802; doi:10.1186/1471-2180-13-68)
Supplement: Additional file 2 — Mini web-site that contains all figures generated in this study. This mini web-site contains all figures of genotype-phenotype, projection and phenotype clustering results. [file 1471-2180-13-68-S2.zip › Bayjanovetal_2012_Lactis/lactisGTleft.html]

1. IL1403 Group1
  
2. IL1403 Group2
  
3. IL1403 Group3
  
4. IL1403 Group4
  
5. IL1403 Group5
  
6. SK11 Group1
  
7. SK11 Group2
  
8. SK11 Group3
  
9. SK11 Group4
  
10. SK11 Group5
  
11. MG1363 Group1
  
12. MG1363 Group2
  
13. MG1363 Group3
  
14. MG1363 Group4
  
15. MG1363 Group5
  
16. KF147 Group1
  
17. KF147 Group2
  
18. KF147 Group3
  
19. KF147 Group4
  
20. KF147 Group5
  
