# Supplementary material for: Genotype-phenotype matching analysis of 38 Lactococcus lactis strains using random forest methods
Source: BMC Microbiol. 2013 Mar 26;13:68. doi: 10.1186/1471-2180-13-68 (PMC3637802; doi:10.1186/1471-2180-13-68)
Supplement: Additional file 2 — Mini web-site that contains all figures generated in this study. This mini web-site contains all figures of genotype-phenotype, projection and phenotype clustering results. [file 1471-2180-13-68-S2.zip › Bayjanovetal_2012_Lactis/lactisPhenoClustMain.html]

Phenotype-based clustering plots
