# Supplementary material for: Genotype-phenotype matching analysis of 38 Lactococcus lactis strains using random forest methods
Source: BMC Microbiol. 2013 Mar 26;13:68. doi: 10.1186/1471-2180-13-68 (PMC3637802; doi:10.1186/1471-2180-13-68)
Supplement: Additional file 2 — Mini web-site that contains all figures generated in this study. This mini web-site contains all figures of genotype-phenotype, projection and phenotype clustering results. [file 1471-2180-13-68-S2.zip › Bayjanovetal_2012_Lactis/lactisProjectLeft.html]

1. E34 DRA4 Li-1 KF7
  
2. FG2 LMG14418 P7304 P7266
  
3. K231 UC317 NCDO895 LMG8520
  
4. KF146 LMG9449 KF147 SK11 MG1363 IL1403
  
5. KF196 KF201 LMG9446 NCDO763
  
6. KF24 AM2 N42 N41
  
7. KF282 LMG6897T M20 KW10
  
8. KF67 V4 ATCC19435T HP
  
9. LMG8526 KF134 K337 ML8
  
